# Investigating the Outcomes and Complications of Urethroplasty Using Different Graft Materials in Men With Complex or Recurrent Urethral Strictures

**DOI:** 10.7759/cureus.86119

**Published:** 2025-06-16

**Authors:** Muhammad Umer Khan, Muhammad Dawood, Muhammad Abu Darda Malhi, Muhammad Haroon Haider, Ali Hussain, Azfar Ali, Samreen Qureshi

**Affiliations:** 1 Surgery, District Headquarter Hospital, Sheikhupura, Sheikhupura, PAK; 2 Surgery, Tehsil Headquarter Hospital, Ferozewala, Ferozewala, PAK; 3 Surgery, Allama Iqbal Teaching Hospital, Dera Ghazi Khan, Dera Ghazi Khan, PAK; 4 Urology, Nishtar Hospital, Multan, PAK; 5 Urology, Multan Institute of Kidney Diseases, Multan, PAK; 6 Urology and Kidney Transplantation, Ameer-ud-Din Medical College, PGMI, Lahore General Hospital, Lahore, PAK; 7 Surgery, Continental Medical College, Lahore, PAK

**Keywords:** buccal mucosa, graft, penile skin, urethral strictures, urethroplasty

## Abstract

Background

Complex and recurrent urethral strictures present significant surgical challenges, with graft-based urethroplasty being the mainstay of treatment. Optimal graft selection remains controversial, particularly between buccal mucosa and penile skin grafts.

Objective

To compare clinical outcomes, complication rates, and patient-reported satisfaction following urethroplasty using buccal mucosa versus penile skin grafts.

Methods

This retrospective cohort study was conducted at Nishtar Hospital, Multan, Pakistan, from January 2022 to December 2024. A total of 178 male patients who underwent substitution urethroplasty were included in the study. Data were retrospectively extracted from electronic medical records and surgical logs. Collected variables included patient demographics (age, comorbid conditions like diabetes or smoking status), stricture characteristics (location, length, etiology, previous treatments), surgical details (graft type, operative time, perioperative complications), and postoperative outcomes. Data were analyzed using SPSS Version 26.0 (IBM SPSS Statistics for Windows, IBM Corp., Armonk, NY). Continuous variables were compared using t-tests and categorical variables using chi-square tests, with p < 0.05 considered statistically significant.

Results

Surgical success rates were higher in the buccal mucosa group at both 12 months (91 (87.5%) vs. 59 (80.2%)) and 24 months (88 (84.6%) vs. 56 (75.6%)), though differences were not statistically significant. Stricture recurrence and graft contracture were lower in the buccal mucosa group (11 (10.6%) vs. 14 (18.9%)) and (four (3.8%) vs. eight (10.8%)), respectively. International Prostate Symptom Score (IPSS) improvement was significantly greater with buccal mucosa grafts (p = 0.04), and patient satisfaction rates were also higher. Complication rates were low in both groups, but oral donor site morbidity was observed in 19 (18%) of buccal mucosa patients.

Conclusions

It is concluded that buccal mucosa grafts offer superior functional outcomes, lower complication rates, and greater patient satisfaction compared to penile skin grafts in the setting of complex or recurrent urethral strictures. Buccal mucosa should be considered the preferred graft material when feasible.

## Introduction

Urethral stricture disease remains a significant urological challenge, particularly when it presents as complex or recurrent cases. Surgical reconstruction, specifically urethroplasty, has long been established as the gold standard for definitive management of these strictures. Graft material selection for urethroplasty directly affects procedure outcomes at both immediate and lasting assessment periods [1]. Buccal mucosa and penile skin show strong popularity as graft options because they possess beneficial biological characteristics, including abundant blood supply, and match well with moist environments as well as straightforward harvesting techniques [2]. Although these grafts are widely used, reports on their success, functional outcomes, and complications vary. Treatment decisions are further complicated by factors such as the location and length of the stricture, past procedures, and patient-specific characteristics [3]. Buccal mucosa grafts (BMGs) serve as the preferred choice because they resist both infection and hair growth, but penile skin grafts (PSGs) show specific benefits in patients whose oral health is at stake [4].

A comprehensive review of different graft materials must happen for recurrent strictures treatment because the complexity of these cases, alongside rising re-operative needs, requires absolute assessment of outcomes. The evaluation of graft survival rates with repeat operations and patient satisfaction indexes, together with recurrence, fistula, and contracture complications, helps advance patient care practices [5]. This study conducts a comprehensive evaluation of health results together with complications that arise from buccal mucosa and PSG implementation during urethroplasty procedures aimed at treating complex or recurrent urethral strictures in male patients [6]. Surgical treatment of complex and recurrent strictures poses distinct challenges because they typically exhibit severe fibrosis together with multiple previous surgeries and inadequate blood supply to the area where the new urethra will be created [7]. Such factors reduce the treatment effectiveness of simple dilation and endoscopic procedures, so additional complex reconstructive techniques become necessary. Medical professionals use graft-based urethroplasty with substitution urethroplasty as an established technique to reconstruct urethras when treating complex cases [8]. The inner lining of the cheek and lip provides BMGs, which medical practitioners widely use for urethral reconstruction because this tissue possesses essential beneficial properties. When placed into the urethra, the tissue shows early development of blood vessels while maintaining its usual moisture preference as well as thick epithelium properties. The success rates of buccal graft urethroplasty continue to rise in complex anatomical conditions based on clinical evidence [9]. The significant adverse consequences of donor site morbidity encompass oral discomfort and numbness, together with restricted mouth opening, which affect the selection process for grafts, mainly when patients suffer from existing oral pathology. The benefits and drawbacks of PSGs derived from inner preputial and distal penile skin for replacement options have been identified [10]. The bare skin on the penis provides accessible tissue that shows similar elasticity and texture to native urethral epithelium while remaining without hair. The lack of oral morbidity resulting from PSGs comes with complications that involve graft contraction and donor-site cosmetic discontentment. Genital skin becomes an inappropriate procedure when used for patients who present with lichen sclerosis or other penile skin diseases due to potential contraindications [11]. Buccal mucosa and PSGs are chosen based on technical aspects of the surgery, which determine the use of dorsal or ventral onlay techniques and extend across various stricture lengths while determining single- or multi-stage operative procedures. The phalloplasty landscape becomes complex because new techniques involving lingual mucosa grafting and tissue engineering with synthetic scaffolds exist, but these experimental methods yield less established results than classic penile and buccal skin grafts [12].

This study aims to compare the clinical outcomes, complication rates, and patient-reported satisfaction following urethroplasty using buccal mucosa versus PSGs.

## Materials and methods

Methodology

This retrospective cohort study was conducted at Nishtar Hospital, Multan, Pakistan, from January 2022 to December 2024. A total of 178 male patients who underwent substitution urethroplasty were included in the study. This study was conducted in accordance with the principles outlined in the Declaration of Helsinki. Ethical approval was obtained from the Institutional Review Board (IRB) of the institute. Given the retrospective nature of the study, the requirement for informed consent was waived by the IRB. All patient data were anonymized and handled with strict confidentiality. The manuscript has been prepared in compliance with the STROBE (Strengthening the Reporting of Observational Studies in Epidemiology) guidelines.

Participant's selection

The study included patients aged 18 years or older who were diagnosed with complex (defined as stricture length greater than 2 cm, involvement of multiple sites, or presence of extensive fibrosis) or recurrent anterior urethral strictures. All included patients had undergone urethroplasty using either buccal mucosa or PSGs.

**Figure 1:**
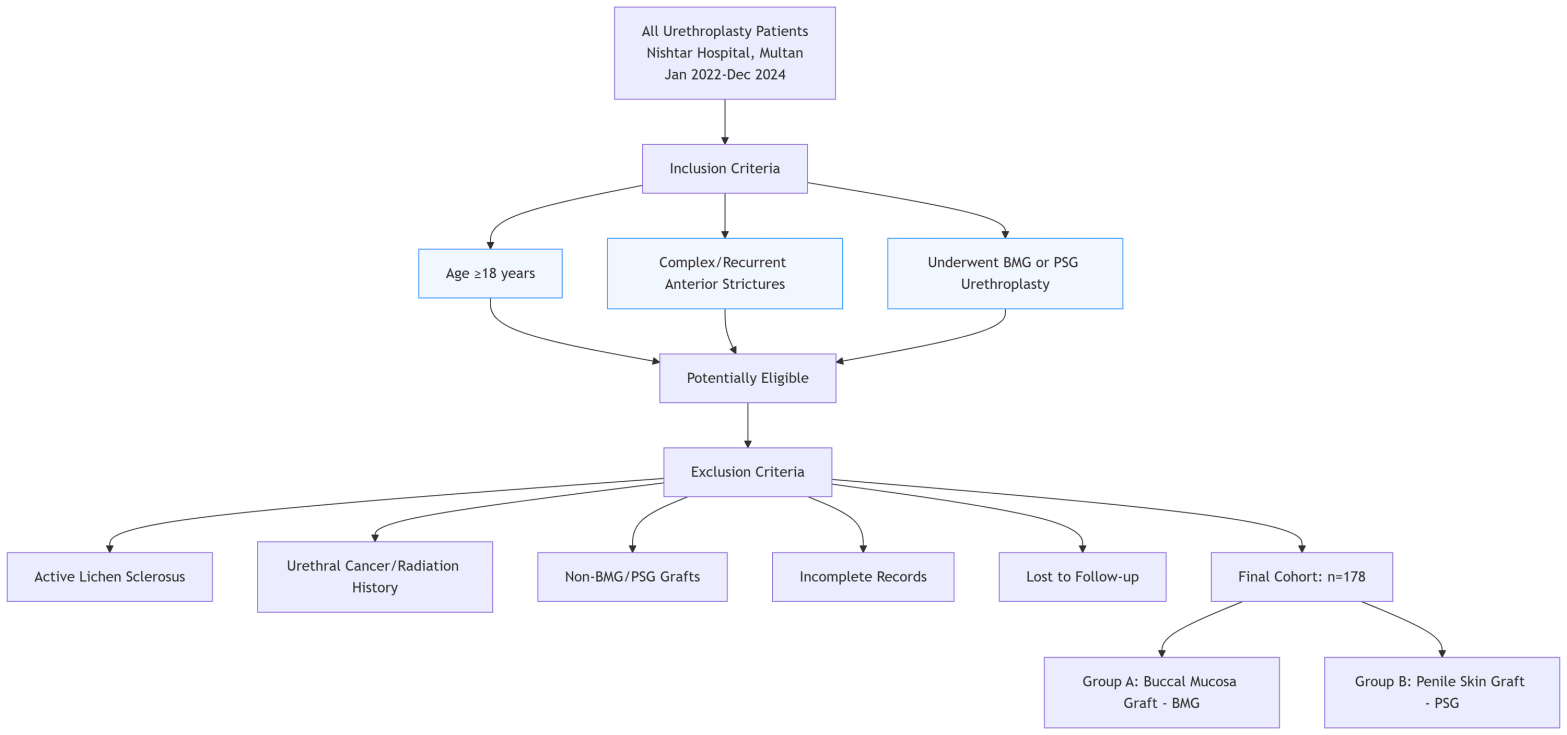
Participant Selection Flowchart

Patients were excluded if their strictures were secondary to active lichen sclerosus, if they had a history of urethral cancer or pelvic radiation therapy, or if grafts other than buccal mucosa or penile skin were used. Additionally, patients with incomplete medical records or those lost to follow-up were excluded from the study (Figure 1).

Data collection

Data were retrospectively extracted from electronic medical records and surgical logs. Collected variables included patient demographics (age, comorbid conditions like diabetes or smoking status), stricture characteristics (location, length, etiology, previous treatments), surgical details (graft type, operative time, perioperative complications), and postoperative outcomes. Based on the graft material used, the patients were categorized into two groups. Group A consisted of those who underwent BMG urethroplasty, while Group B comprised patients who received PSG urethroplasty. All urethroplasty procedures were performed by experienced reconstructive urologists using standardized surgical techniques. Different stricture characteristics dictated whether surgeons chose dorsal or ventral onlay approaches for treatment. BMGs received their source material from the inner cheek area during the procedure, where hemostasis requirements received priority, along with an emphasis on limited donor site complications. Skin grafts were retrieved from hairless surfaces of the prepuce along with low portions of the penile shaft to guarantee the best tissue quality. Surgeon skill was matched with patient wishes to decide between using BMG or PSG, while specific clinical considerations involving oral and genital pathology also played a role in the selection process. Healthcare professionals performed tests that included uroflowmetry for studying urinary speeds and post-void residual urine measurements, as well as questionnaire evaluations for measuring patient contentment. Every complication during both the early and late periods post-surgery, including bleeding, infection, wound dehiscence, stricture recurrence, graft contracture, and fistula formation, was documented.

Data analysis

Statistical analyses were conducted using SPSS Version 26.0 (IBM SPSS Statistics for Windows, IBM Corp., Armonk, NY). Continuous variables (e.g., age, stricture length, Qmax (maximum flow rates) improvement, International Prostate Symptom Scores (IPSS) improvement) are presented as means ± standard deviations and were compared between groups using independent Student’s t-tests. Categorical variables (e.g., diabetes prevalence, surgical success rates, complication frequencies) are reported as proportions (%) and analyzed using Pearson’s chi-square test. Surgical success and recurrence rates at 12- and 24-month follow-ups were compared using chi-square tests for proportions at each time point. Statistical significance was defined as a p-value of less than 0.05. Patient-reported outcomes (e.g., satisfaction, donor site morbidity) were analyzed descriptively or with chi-square/t-tests where appropriate.

## Results

A total of 178 patients were included, with 104 patients undergoing BMG (Group A) and 74 patients receiving PSGs (Group B). The mean age was comparable between the groups (49.6 ± 12.7 vs. 51.1 ± 13.2 years; p = 0.42). There were no significant differences regarding the prevalence of diabetes (23 (22%) vs. 18 (24%); p = 0.71), smoking history (32 (31%) vs. 21 (29%); p = 0.83), or previous urethral surgery (47 (45%) vs. 36 (48%); p = 0.67) (Table 1).

**Table 1:** Baseline Patient Demographics

Characteristic	Group A (Buccal Mucosa)	Group B (Penile Skin)	p-value	Test Statistic	Effect Size
Number of patients	104	74	-	-	-
Mean Age (Years)	49.6 ± 12.7	51.1 ± 13.2	0.42	t(176) = -0.81	d = -0.12
Diabetes Mellitus (%)	23 (22%)	18 (24%)	0.71	χ²(1) = 0.14	φ = 0.028
Smoking History (%)	32 (31%)	21 (29%)	0.83	χ²(1) = 0.045	φ = 0.016
Previous Urethral Surgery (%)	47 (45%)	36 (48%)	0.67	χ²(1) = 0.19	φ = 0.033

At 12 months, the surgical success rate was 91 (87.5%) in the buccal mucosa group and 59 (80.2%) in the penile skin group (p = 0.18), with similar trends observed at 24 months (88 (84.6%) vs. 56 (75.6%); p = 0.12). Stricture recurrence was lower in Group A (11 (10.6%)) compared to Group B (14 (18.9%); p = 0.09), while graft contracture occurred less frequently in Group A (four (3.8%) vs. eight (10.8%); p = 0.05). Patient satisfaction rates were high in both groups (88 (85%) vs. 58 (78%); p = 0.21), and Qmax improvement was significantly greater in the buccal mucosa group (+12.1 mL/second vs. +10.6 mL/second; p < 0.001) (Table 2).

**Table 2:** Comparison of Clinical Outcomes Between Buccal Mucosa and Penile Skin Grafts Qmax, Maximum Flow Rate p-values < 0.05 were considered statistically significant.

Outcome	Group A (%)	Group B (%)	p-value	Test Statistic	Effect Size
Surgical Success (12 Months)	91 (87.5%)	59 (80.2%)	0.18	χ²(1) = 1.80	φ = 0.101
Surgical Success (24 Months)	88 (84.6%)	56 (75.6%)	0.12	χ²(1) = 2.48	φ = 0.118
Stricture Recurrence	11 (10.6%)	14 (18.9%)	0.09	χ²(1) = 2.82	φ = 0.126
Graft Contracture	4 (3.8%)	8 (10.8%)	0.049	χ²(1) = 3.89	φ = 0.148
Patient Satisfaction	88 (85%)	58 (78%)	0.21	χ²(1) = 1.55	φ = 0.094
Qmax Improvement (mL/s)	12.1	10.6	<0.001	t(176) = 5.07	d = 0.42

Wound infection rates were comparable (five (5%) in Group A vs. five (7%) in Group B; p = 0.56), as were hematoma occurrences (two (2%) vs. two (3%); p = 0.72). Oral donor site morbidity was reported in 19 (18%) of patients in the buccal mucosa group. Fistula formation was slightly higher in the penile skin group (three (4.0%) vs. two (1.9%); p = 0.41), and graft contracture was significantly more common in Group B (eight (10.8%) vs. four (3.8%); p = 0.05) (Table 3).

**Table 3:** Postoperative Complications in Buccal Mucosa and Penile Skin Graft Urethroplasty p-values < 0.05 were considered statistically significant.

Complication	Group A (%)	Group B (%)	p-value	χ² (df = 1)	Cramér’s V
Wound Infection	5 (5%)	5 (7%)	0.56	0.34	0.044
Hematoma	2 (2%)	2 (3%)	0.72	0.13	0.027
Oral Donor Site Morbidity	19 (18%)	N/A	-	-	-
Fistula Formation	2 (1.9%)	3 (4%)	0.41	0.68	0.062
Graft Contracture	4 (3.8%)	8 (10.8%)	0.049	3.89	0.148

The mean stricture length was comparable between groups (4.2 ± 1.5 cm in Group A vs. 4.5 ± 1.7 cm in Group B; p = 0.27). Most strictures were located in the bulbar urethra (70 (67%) in Group A vs. 47 (64%) in Group B; p = 0.68), followed by the penile urethra (29 (28%) vs. 22 (30%); p = 0.74). Panurethral involvement was rare (five (5%) vs. four (6%); p = 0.81), and there were no significant differences in stricture etiology, with idiopathic, traumatic, and infectious causes similarly distributed between groups (Table 4).

**Table 4:** Stricture Characteristics p-values < 0.05 were considered statistically significant.

Characteristic	Group A (%)	Group B (%)	p-value	Test Statistic	Effect Size
Mean Stricture Length (cm)	4.2 ± 1.5	4.5 ± 1.7	0.27	t(176) = -1.11	d = -0.18
Bulbar Location	70 (67%)	47 (64%)	0.68	χ²(1) = 0.17	φ = 0.031
Penile Location	29 (28%)	22 (30%)	0.74	χ²(1) = 0.11	φ = 0.025
Panurethral Involvement	5 (5%)	4 (6%)	0.81	χ²(1) = 0.06	φ = 0.018
Idiopathic Etiology	54 (52%)	36 (49%)	0.72	χ²(1) = 0.13	φ = 0.027
Traumatic Etiology	30 (29%)	23 (31%)	0.77	χ²(1) = 0.08	φ = 0.021
Infectious Etiology	20 (19%)	15 (20%)	0.85	χ²(1) = 0.07	φ = 0.020

Postoperative patient-reported outcomes showed greater IPSS improvement in the buccal mucosa group compared to the penile skin group (13.2 ± 3.5 vs. 11.8 ± 4.1; p = 0.04). Patient satisfaction was high in both groups (88 (85%) in Group A vs. 58 (78%) in Group B; p = 0.21). Donor site morbidity was reported in 19 (18%) of patients undergoing BMG harvesting. Rates of sexual function worsening were low and similar between groups (five (5%) vs. four (6%); p = 0.79) (Table 5).

**Table 5:** Patient Reported Outcomes (PROMs) IPSS, International Prostate Symptom Score p-values < 0.05 were considered statistically significant.

Parameter	Group A	Group B	p-value	Test Statistic	Effect Size
IPSS Improvement	13.2 ± 3.5	11.8 ± 4.1	0.04	t(176) = 2.06	d = 0.37
Patient Satisfaction (%)	88 (85%)	58 (78%)	0.21	χ²(1) = 1.55	φ = 0.094
Donor Site Morbidity	19 (18%)	N/A	-	-	-
Sexual Function Worsening	5 (5%)	4 (6%)	0.79	χ²(1) = 0.07	φ = 0.020

## Discussion

In this retrospective cohort study, we compared the outcomes and complications of urethroplasty using buccal mucosa and PSGs in men with complex or recurrent urethral strictures. Our research shows BMGs delivered superior functional results with fewer complications and better patient satisfaction than PSGs, though most outcomes remained statistically insignificant. The clinical success rate scores for BMGs reached 91 (87.5%) at 12 months and 88 (84.6%) at 24 months, which outperformed PSGs to 59 (80.2%) and 56 (75.6%), respectively. These results match published research about the superiority of buccal graft urethroplasty in complex surgical conditions. Patient-specific factors determine which graft type is optimal despite the reported trends, as the statistical differences did not reach significance [13]. The incidence of stricture recurrence proved lower for patients who received BMGs versus those with other methods (11 (10.6%) vs. 14 (18.9%)), and graft contracture occurred less frequently in buccal mucosa patients (four (3.8%) vs. eight (10.8%)). The identification of these findings holds great value because graft contracture combined with stricture recurrence will most likely result in further operations that could diminish patient quality of life. The superior vascular flow and strong resistance to mechanical stress of buccal mucosa possibly explain its lower complication rate after placement inside the urethra [14]. BMGs provided superior patient-reported results compared to PSGs according to IPSS score evaluations (13.2 ± 3.5 vs. 11.8 ± 4.1). The patient satisfaction evaluation also leaned toward favoring buccal mucosa, but the difference was not statistically significant (88 (85%) vs. 58 (78%)). The adverse impact on the donor sites was found to be low since 18% of patients who underwent buccal mucosa procedures developed temporary oral discomfort that surgeons could easily manage. Research showed matching sexual function results between the groups, indicating the donor choice did not result in significant modifications of postoperative erectile function [15]. Studies revealed wound infection and hematoma occurred with equal frequency between groups without statistical significance. The use of PSGs led to fistula formation in three (4.0%) of cases compared to two (1.9%) in the other groups, but PSGs exhibited higher graft-related complications such as contraction [16]. Careful patient selection remains crucial prior to using genital skin in substitution urethroplasty because patients with penile skin conditions or weak tissue must not be candidates for this approach. The stricture quality measures remained alike between populations despite most patients having bulbar urethra strictures of 4-4.5 cm length [17]. A comparable assessment of stricture causes provided balanced study results since the comparison between groups included no meaningful confounding elements. Several weaknesses exist in this research because it relies on retrospective data collection and because the assessment of late recurrences extends only up to two years. The weak power to establish statistical significance in certain outcome measures could have occurred because of the sample size limitation, which specifically afflicted the penile skin group. Long-term data from prospective randomized controlled studies should be collected to determine the best tissue option for treating complex and recurrent urethral strictures.

## Conclusions

It is concluded that both buccal mucosa and PSGs are effective materials for substitution urethroplasty in men with complex or recurrent urethral strictures. However, BMGs demonstrated slightly higher surgical success rates, better postoperative urinary function, and lower graft-related complications compared to PSGs. Patient satisfaction was also greater with buccal mucosa, despite minor donor site morbidity.

## References

[1] Reichert M, Aragona M, Soukkar A, Olianas R (2022). Mesh graft urethroplasty—still a safe and promising technique in mostly unpromising complex urethral strictures. J Clin Med.

[2] Frankiewicz M, Markiet K, Krukowski J, Szurowska E, Matuszewski M (2021). MRI in patients with urethral stricture: a systematic review. Diagn Interv Radiol.

[3] Pandian RM, John NT, Eapen A, Antonisamy B, Devasia A, Kekre N (2017). Does MRI help in the pre - operative evaluation of pelvic fracture urethral distraction defect? - a pilot study. Int Braz J Urol.

[4] Gelman J, Wisenbaugh ES (2015). Posterior urethral strictures. Adv Urol.

[5] Barbagli G, Palminteri E, Guazzoni G, Montorsi F, Turini D, Lazzeri M (2005). Bulbar urethroplasty using buccal mucosa grafts placed on the ventral, dorsal or lateral surface of the urethra: are results affected by the surgical technique?. J Urol.

[6] Myers JB, Brant WO, Hotaling JN, Lenherr SM (2017). Use of alternative techniques and grafts in urethroplasty. Urol Clin North Am.

[7] Liang CZ, Zhang XJ, Hao ZY, Yang S, Wang DB, Shi HQ, Liu C (2004). An epidemiological study of patients with chronic prostatitis. BJU Int.

[8] Barbagli G, Akbarov I, Heidenreich A (2018). Anterior urethroplasty using a new tissue engineered oral mucosa graft: surgical techniques and outcomes. J Urol.

[9] Webster GD, Sihelnik S (1985). The management of strictures of the membranous urethra. J Urol.

[10] Bischoff R, Marcon J, Schulz GB (2025). Perioperative outcomes and trends of surgical correction of male urethral strictures: results from the GRAND study. J Clin Med.

[11] Vashishta S, Sureka AK, Kumar J (2014). Predictors for recurrence after urethroplasty in pediatric and adolescent stricture urethra. J Pediatr Urol.

[12] Cannoletta D, Pederzoli F, Yepes C (2025). Evolution and innovation in urethroplasty: a comprehensive narrative review of graft types and surgical techniques. Int J Impot Res.

[13] Spilotros M, Sihra N, Malde S, Pakzad MH, Hamid R, Ockrim JL, Greenwell TJ (2017). Buccal mucosal graft urethroplasty in men-risk factors for recurrence and complications: a third referral centre experience in anterior urethroplasty using buccal mucosal graft. Transl Androl Urol.

[14] Favorito LA, Conte PP, Sobrinho UG, Martins RG, Accioly T (2018). Double inlay plus ventral onlay buccal mucosa graft for simultaneous penile and bulbar urethral stricture. Int Braz J Urol.

[15] Marks P, Dahlem R, Janisch F (2023). Mucomucosal anastomotic non-transecting augmentation (MANTA) urethroplasty: a ventral modification for obliterative strictures. BJU Int.

[16] Abbasi B, Shaw NM, Lui JL (2022). Comparative review of the guidelines for anterior urethral stricture. World J Urol.

[17] Bogdanov AB, Veliev EI, Sokolov EA (2021). Nontransecting anastomotic urethroplasty via ventral approach without full mobilization of the corpus spongiosum dorsal semicircumference. Urology.

